# Preventing the Complications Associated with the Use of Dermal Fillers in Facial Aesthetic Procedures: An Expert Group Consensus Report

**DOI:** 10.1007/s00266-017-0798-y

**Published:** 2017-04-14

**Authors:** Fernando Urdiales-Gálvez, Nuria Escoda Delgado, Vitor Figueiredo, José V. Lajo-Plaza, Mar Mira, Francisco Ortíz-Martí, Rosa del Rio-Reyes, Nazaret Romero-Álvarez, Sofía Ruiz del Cueto, María A. Segurado, Cristina Villanueva Rebenaque

**Affiliations:** 1 Instituto Médico Miramar, Paseo de Miramar 21, 29016 Málaga, Spain; 2 Centro de Medicina Estética Dra Escoda, Rambla de Catalunya 60, Barcelona, Spain; 3 Clínica Milénio, R. Manuel da Silva Leal 11C, Lisbon, Portugal; 4 Centro Médico Lajo-Plaza, Calle Moreto 10, Madrid, Spain; 5 Clínica Mira + Cueto, Av. de Concha Espina 53, Madrid, Spain; 6 Teknobell Médicina Estética, Av. Pdte Carrero Blanco 14, Seville, Spain; 7 Grupo de Dermatología Pedro Jaén, Calle Cinca 30, Madrid, Spain; 8 Clínica de Medicina Estética Dra Nazaret Romero, Paseo Castellana 123, Madrid, Spain; 9 SClinic, Claudio Coello 92, Madrid, Spain; 10 Hospital del Sureste Vía Verde, Ronda del Sur 10, Arganda del Rey, Madrid, Spain; 11 Clínica de Medicina Estética Dra Villanueva, Carrer de Calvet 10, Barcelona, Spain

**Keywords:** Aesthetic procedures, Dermal fillers, Complications, Prevention

## Abstract

**Background:**

The use of dermal fillers in minimally invasive facial aesthetic procedures has become increasingly popular of late, yet as the indications and the number of procedures performed increase, the number of complications is also likely to increase. Paying special attention to specific patient characteristics and to the technique used can do much to avoid these complications. Indeed, a well-trained physician can also minimize the impact of such problems when they do occur.

**Methods:**

A multidisciplinary group of experts in aesthetic treatments reviewed the main factors associated with the complications that arise when using dermal fillers. A search of English, French and Spanish language articles in PubMed was performed using the terms “complications” OR “soft filler complications” OR “injectable complications” AND “dermal fillers”. An initial document was drafted that reflected the complications identified and recommendations as to how they should be handled. This document was then reviewed and modified by the expert panel, until a final text was agreed upon and validated.

**Results:**

The panel addressed consensus recommendations about the preparation, the procedure and the post-procedural care. The panel considered it crucial to obtain an accurate medical history to prevent potential complications. An additional clinical assessment, including standardized photography, is also crucial to evaluate the outcomes and prevent potential complications. Furthermore, the state of the operating theatre, the patient’s health status and the preparation of the skin are critical to prevent superficial soft tissue infections. Finally, selecting the appropriate technique, based on the physician’s experience, as well as the characteristics of the patient and filler, helps to ensure successful outcomes and limits the complications.

**Conclusions:**

This consensus document provides key elements to help clinicians who are starting to use dermal fillers to employ standard procedures and to understand how best to prevent potential complications of the treatment.

**Level of Evidence V:**

This journal requires that authors assign a level of evidence to each article. For a full description of these Evidence-Based Medicine ratings, please refer to the Table of Contents or the online Instructions to Authors www.springer.com/00266.

## Introduction

In recent years, the popularity of minimally invasive cosmetic procedures has experienced unprecedented growth, including the use of dermal fillers. According to the American Society for Aesthetic Plastic Surgery, the use of cosmetic surgery increased nearly 5% between 2011 and 2012 [1]. As the use of dermal fillers becomes more established, the size of the market grows and there are now an estimated 160 products currently available worldwide, supplied by more than 50 companies [2]. These products are mainly used to create volume or to reverse any soft tissue loss due to disease or age [3]. As such, they are used in volume replacement and enhancement procedures that include cheek and chin augmentation, tear trough correction, nose reshaping, mid-facial volumization, lip enhancement, hand rejuvenation and the correction of facial asymmetry [3].

Dermal fillers vary in their composition, duration of effect, palpability, ease of administration, potential complications and other factors, all of which affect the therapeutic results [4, 5]. Hence, achieving desirable outcomes with dermal fillers depends critically on understanding their different characteristics, capabilities, methods of injection, risks and the limitations of the fillers available. In addition, there is a learning curve associated with the administration of dermal fillers, which requires practice to achieve consistently desirable results. Perhaps the most important issue to prevent complications with dermal fillers, more than selecting the appropriate patients, is probably not to treat inappropriate ones.

Because of the large number of dermal fillers currently available in the market and their increasing popularity, not only among patients but also among dermatologists and aesthetic physicians, it is of interest to provide recommendations that might help clinicians in their decision-making. Additionally, the lack of randomized control trials underlines the need to gather consensus views from experienced injectors who have treated many patients.

This manuscript aims to provide a summary of the different factors that might influence the results of dermal fillers in aesthetic procedures and how to prevent them.

Thus, in this article we establish consensus-based recommendations to provide dermatologists and practitioners of aesthetic medicine a reference framework based on available data, and the group’s clinical experience.

## Materials and Methods

On 21 May 2016, a multidisciplinary group of experts in aesthetic treatments convened to discuss the main factors that influence the complications associated with dermal filler use. Different subjects emerged as core topics of concerns including patient selection, injection technique and post-procedural cares. The authors developed this consensus paper based on those discussions and a review of the current literature.

A PubMed literature search for English, French and Spanish language articles published to date was performed using the terms “complications” OR “soft filler complications” OR “injectable complications” AND “dermal fillers”. References cited in selected articles were also reviewed to identify additional relevant reports. Additionally, relevant published national and international guidelines were also scrutinized.

Because the aesthetic procedures are usually elective processes, clinical trials are complex to organize and conduct. There are few prospective trials, but these are often not randomized or controlled. Therefore, our knowledge base mainly comprises case reports and summaries of individual practitioner’s experience.

**Fig. 1:**
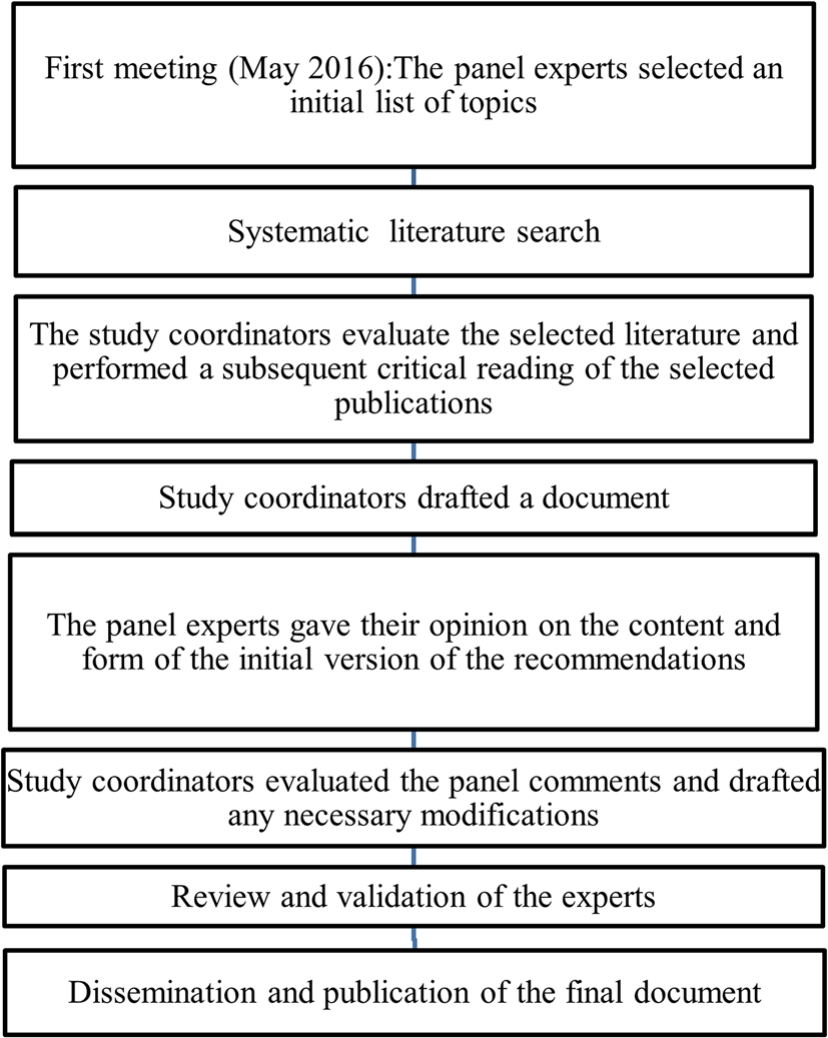
Flow diagram of the consensus process

An initial document was drafted by the Coordinating Committee, and it was reviewed by the expert panel members. The Coordinating Committee evaluated the panel’s comments and modified the draft as they considered necessary. Subsequent revisions were based on feedback from the other authors until a consensus was achieved, and the final text was then validated (Fig. 1).

## Results

### Pre-procedure Care

#### How the Skin Ages: Facial Ageing

Facial ageing is a multifactorial, complex, three-dimensional (3D), dynamic and generally not uniform process, with anatomical, biochemical and genetic correlates [6–8]. Many of the facial manifestations of ageing reflect the combined effects of gravity, progressive bone resorption, decreased tissue elasticity and the redistribution of subcutaneous fullness [6–8]. Facial ageing is also associated with the loss of soft tissue fullness in certain areas (periorbital, forehead, malar, temporal, mandibular, mental, glabella and perioral sites) and the persistence or hypertrophy of fat in others (submental, lateral nasolabial fold, labiomental crease, jowls, infraorbital fat pouches and malar fat pad) [6, 9]. All people age differently as a result of the imbalance, disharmony and disproportion of the ageing process between the overlying soft tissue and the underlying bony frameworks. Moreover, each individual compartment ages at a different pace in the same individual. Hence, it is recommended that when correcting the ageing face, the individual compartments should be considered and corrected separately.

#### Knowledge of the Fillers

There are a host of fillers currently available in Spain and Portugal. Before and after opening the filler, it is imperative to be aware of their constituents, particle concentration (per mg), cross-linking, monophasic or biphasic nature, additives (e.g. lidocaine) and shelf life. Any contraindications detailed in the instructions for the use of the chosen filler should be closely adhered to. The suitability of different fillers needs to be discussed, and the patient must be given an indication of the likely value expected to be gained from the treatment [10, 11]. Although the panel considers that the perfect filler has yet to be described, the characteristics of an optimal filler are listed in Table 1. The choice of fillers depends upon diverse factors, such as the defect to be corrected, the desired duration of the effect and the constituents of the filler.

**Table 1:** Optimal dermal filler characteristics

Dermal filler characteristics check list	
Long duration/persistence Performance promise Painless Reasonable cost Non toxic Non inflammatory Non carcinogenic No migration	Non-animal origin Easy to store Easy to inject Easy of learning how to inject Minimal side effects Biodegradable (in case of temporary or semi-permanent fillers) Biocompatibility

#### Injection Technique and Injection Patterns

Selecting the appropriate injection technique for each patient helps to ensure a successful outcome and limits the risk of undesired effects. The techniques and injection patterns used to administer dermal fillers in clinical practice vary according to physician’s preferences and experience [12]. Several patterns have been described for the appropriate placement of different fillers: fanning, serial puncture, cross-hatching and linear threading. The most popular is the linear threading method, either retrograde or anterograde. Beginners are advised to start with this technique rather than using other methods like the depot or fern method, which can produce lumps if administered inappropriately.

### Preparative Care

#### Patient Interview

Although the grievance the patients express is extremely important, clinicians must be aware that patients may have difficulties in conveying their reasons for seeking advice or treatment. Hence, an exhaustive consultation is essential prior to any treatment to determine the reasons why the patient wants to undergo cosmetic therapy, and to establish “realistic” goals for the treatment. The clinician’s first goal is to determine whether facial ageing exists and, if so, whether the patient’s perception of the deformity ties in with the clinician’s assessment.

The patient’s motivation for undergoing treatment is very important. Patients who have “external” (extrinsic) motivations, for example involving the desire to please others or to have a more successful career, are less likely to be happy with the results of the treatment than patients who have “internal” (intrinsic) motivations and want to look better for themselves [13]. In addition, it is extremely important for patients to have realistic expectations. The goal to look like a top model or the most fashionable film star is, in most cases, not realistic. On the other hand, it is also vital for the clinician to not create unrealistic expectations. The patient must fully understand the results that can be realistically achieved, the approximate length of the treatment and also the likely complications. At this point it is crucial to establish good communications with the patient [13]. Moreover, some prudence should be exercised when deciding about a patient who exhibits signs of any underlying mental disturbance or dysmorphophobic tendency.

#### Medical History

Obtaining a complete medical history is crucial to prevent potential complications. The medical history should scour for any medical illnesses, allergies, medications used and prior aesthetic procedures. For instance, the risk of bruising is greater when patients have bleeding disorders, uncontrolled hypertension or when anticoagulants like aspirin, clopidogrel or warfarin are being taken. Similarly, we must check for hypersensitivity to lidocaine. Anti-ds DNA antibodies cross-react with collagen and, hence, collagen-based fillers should not be used in patients with systemic lupus erythematosus [14]. When evaluating patients with cardiovascular diseases or those who take anticoagulant medication, it is important to determine the time frame during which the patient will be taking the medication and the risk associated with temporary discontinuation of the medication [15].

Although there have only occasionally been reports of adverse events or suboptimal results, caution is advised when injecting hyaluronic acid-based fillers derived from *Streptococcus* species in patients with any prior streptococcal disease. Indeed, this may be a source of delayed reactions [12]. It should be noted that some of the currently available non-animal hyaluronic acid-based fillers contain trace amounts of gram-positive bacterial proteins and, therefore, they are contraindicated for patients with a history of allergies to such material [16]. Finally, it is extremely important to check for any signs of inflammation in the area to be treated. Active inflammation will provoke degradation of the filler [10].

Table 2 summarizes the minimum requirements in terms of the information that should be gleaned from the patient’s medical records for aesthetic procedures.

**Table 2:** Minimum requirements demanded to the medical records in aesthetic procedures

Minimal information collected in the medical record
Patient identification
Number of clinical record
Name and surname
Date of birth
Address
Telephone number
Reason for the visit
Main reason for consultation
Duration of the problem
Family history
Psychosocial history
Patient motivation
Patient expectations
Level of education
Kind of work
Hygiene conditions
Medical history
Allergies*, immunological diseases, herpes
Previous and current diseases
Previous surgeries
Previous aesthetic treatments**
Prior infections (pay special attention on this point as most of filler complications are related to this issue)
Dental history***
Active skin infections or inflammations
Clinical assessment
To determine whether a facial ageing process exists
Well-focused pre-treatment photographs
Assessment of treatment effects
Assessment of adverse effects
Medicolegal purposes
Ultrasonography

* The panel recommends to pay special attention for asking about allergy/hypersensitivity reactions to lidocaine (information may be obtained from previous dental procedures)

** According to the panel recommendations, previous aesthetic procedures must be thoroughly assessed. The physician must be sure about the filler/fillers injected

*** After a dental procedure, such as dental removal, dental cleaning or drilling the panel recommends to wait, at least, 1 month before injecting the fillers

#### Informed Consent

It is mandatory that the patient receives adequate information about the filler, the technique that will be used for its administration, the potential outcomes, the possible side effects, the post-procedural care, the need for maintenance or any other additional procedures required to achieve optimum results.

The panel recommends that this informed consent:

1. Should be as complete as possible, such that it could serve as a guide to obtain and impart all the necessary information at the first visit.
2. Should include a specific paragraph regarding the potential problems when the patient also suffers from an immune disease.

In addition to the informed consent, it is recommended that the patient fills out a questionnaire in which any previous treatments with fillers are recorded, as well as the type of filler and their response.

#### Clinical Assessment

As Claude Bernard said: “Who doesn’t know what he’s looking for, doesn’t understand what he finds”. Every face has its disproportions and asymmetries, and they are essentially normal. Therefore, a clinician must employ an educated eye if the correct diagnosis is to be made. To analyse an individual’s facial proportions, their head position must be natural. A natural head position is defined as a standardized and reproducible position of the head when the subject focuses on a distant point at eye level [13].

**Fig. 2:**
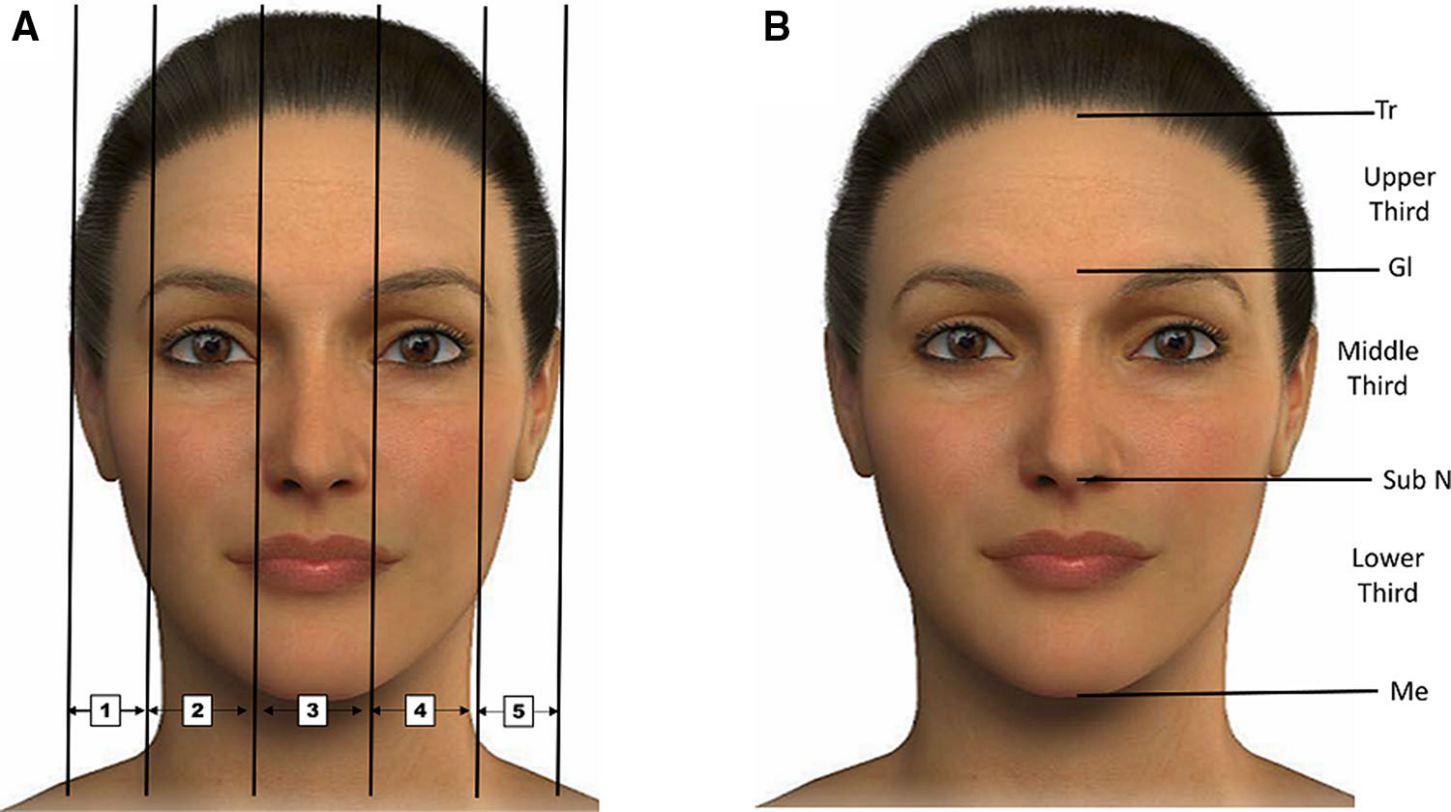
Different divisions of the face. **a** Division of the face into *vertical* fifths. 1: Pa right–Ex right, 2: Ex right–En right, 3: En right–En left, 4: En left–Ex left, 5: Ex left–Pa left. **b** Division of the face into *horizontal* thirds. 1 upper third: Tr–Gl, 2 middle third: Gl–subN, 3 lower third: subN–Me. *Pa* postaurale, *Ex* exocanthion, *En* endocanthion, *Tr* trichion, *Gl* glabella, *Me* menton, *SubN* subnasale

**Fig. 3:**
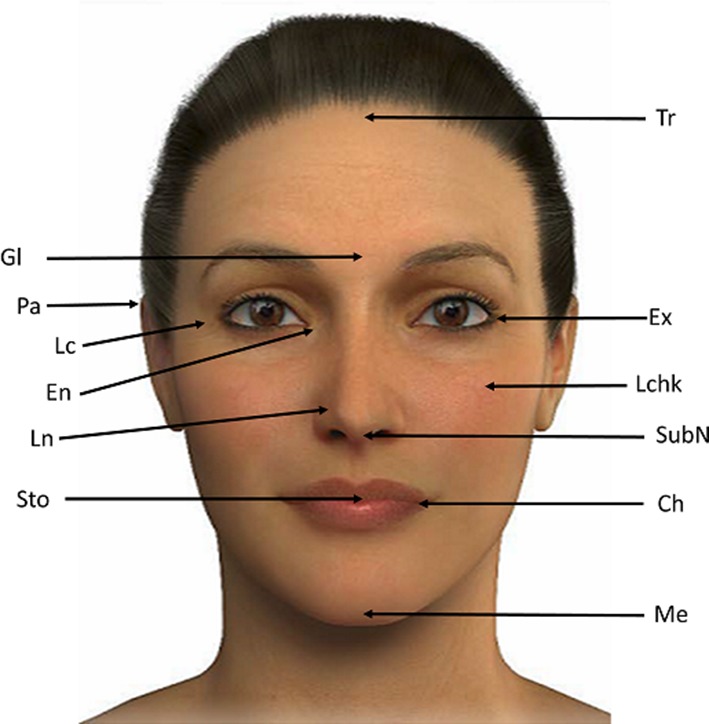
Soft tissue points that can be used to obtain face measurements (adapted from Milutinovic et al. [20]). Soft tissue points from *top* to *bottom*: trichion (Tr): the beginning of the forehead when one lifts the eyebrow, glabella (Gl): the most prominent point of the forehead at the superior aspect of the eyebrows, postaurale (Pa): the most posterior point on the helix (outer rim of the ear), lateral canthus (LC): lateral canthus of the eye, exocanthion (Ex): the most lateral point of the palpebral fissure at the outer canthus of the eye, endocanthion (En): the most medial point of the palpebral fissure at the inner canthus of the eye, lateral cheek (Lchk): lateral border of the cheeks, lateral nose (Ln): lateral side of the nose, subnasale (SubN): the point in the midsagittal plane where the nasal septum merges into the upper lip, stomion (Sto): the midpoint of the intralabial fissure, cheilion (Ch): the corner of the mouth, menton (Me): the most inferior point on the soft tissue chin

**Fig. 4:**
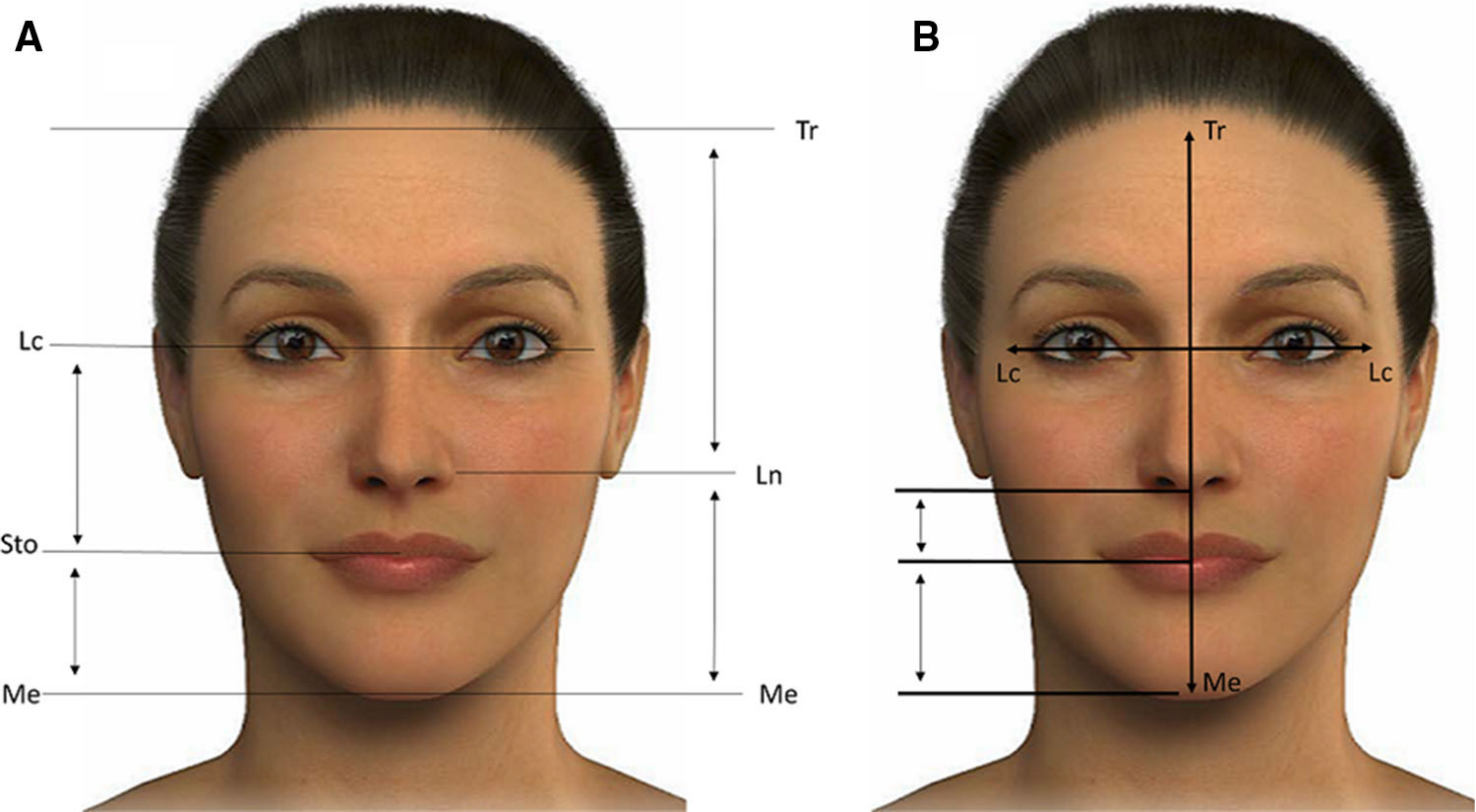
Different parameters to calculate the length of the face (adapted from Milutinovic et al. [20]). Facial measures (Fig. 1a, b): (Tr–Me): height of the face. (Lchk right–Lchk left): width of the face. (Me–Sto): the lowest point on the chin, and the point where the upper and lower lip merge. (Sto–LC): the point where the upper and lower lip merge, and corner of the eye. (Me–Ln): the lowest point on the chin and the outer edge of the nostril. (Ln–Tr): the outer edge of the nostril and highest point of the forehead

Many linear and angular measures of the soft tissue profile, and a variety of cephalometric analyses, have been developed to determine the ideal facial proportions [17–20]. Beauty is not an exact science, but according to some plastic surgeons, there is a specific proportion system that takes into account facial height, width and symmetry. Furthermore, the definition of an attractive and beautiful face is subjective, associated with many factors that include social, cultural and ethnic aspects, as well as age [21]. The face may be divided into vertical fifths (Fig. 2a) and horizontal thirds (Fig. 2b) [20]. To evaluate facial proportions, several soft tissue points can be used to obtain linear distances (Fig. 3) and, in fact, these facial measurements may be strongly related to attractiveness [20, 22] (the different parameters measured to assess beauty are shown in Fig. 4).

The panel members considered it extremely important to evaluate the quality of the skin, assessing the presence of nevus or other skin irregularities that could reflect systemic illnesses or predict potential skin complications after filler injection.

##### Ultrasound Examination

Ultrasonography may be a useful tool to evaluate the surface topography of the skin and to assess what may happen to the filler beneath the surface of the skin [22, 23]. Ultrasonography was used to ascertain the site, quantity and type of filler that should be injected into the soft tissue of the face in a cohort of 80 subjects who underwent facial filler augmentation [24]. High-frequency sonography was able to identify and quantify the presence of the filler in the soft tissue of almost all patients. Moreover, it was possible to detect inflammatory reactions (many of which were silent) and granulomas and to detect the presence of diverse fillers in the same area [24]. Additionally, ultrasonography may facilitate the selection and application of rejuvenation agents and procedures (such as lower eyelid blepharoplasty), the dynamic analysis of hyaluronic acid within the elevator plane for upper eyelid retraction, and the serial distribution and integration of autologous fat injection in the lower lid compartments [25].

##### Standardized Photographic Scales

Among the different methods used to analyse craniofacial morphology and to establish facial profiles, standardized photographs occupy a prominent place in facial analysis and they are used routinely by most aesthetic specialists. Additionally, high‐quality standardized photographs “before and after” provide a compelling demonstration of the possible results of treatment and they are a crucial tool that aid quality consultations [26]. Although scales have been published that permit intra-study comparisons, many of them are yet to be validated, and their heterogeneity makes comparisons across studies impossible. A set of validated, objective and quantitative scales now exists that allows the key signs of the ageing that causes individuals to seek cosmetic procedures to be evaluated [27–32]. Each scale is a five-point photo-numeric scale based on computer-simulated photographs incorporating each of the aspects to be evaluated in a stepwise manner. Other methods have also been proposed as valuable tools in aesthetics, such as the VISIA Complexion Analysis System (Canfield Imaging Systems, Fairfield, NJ) [33] or the Vectra 3D imaging software (Canfield Scientific, Inc. Fairfield, New Jersey) [34]. The Mid-Face Volume Deficit Scale (MVDS) is an Allergan® specific scale that uses a six-point photo-numeric instrument specifically developed as a physician’s assessment tool to evaluate overall degree of the volume deficit of the mid-face, from 0 (none) through to 6 or severe (Fig. 4).

**Fig. 5:**
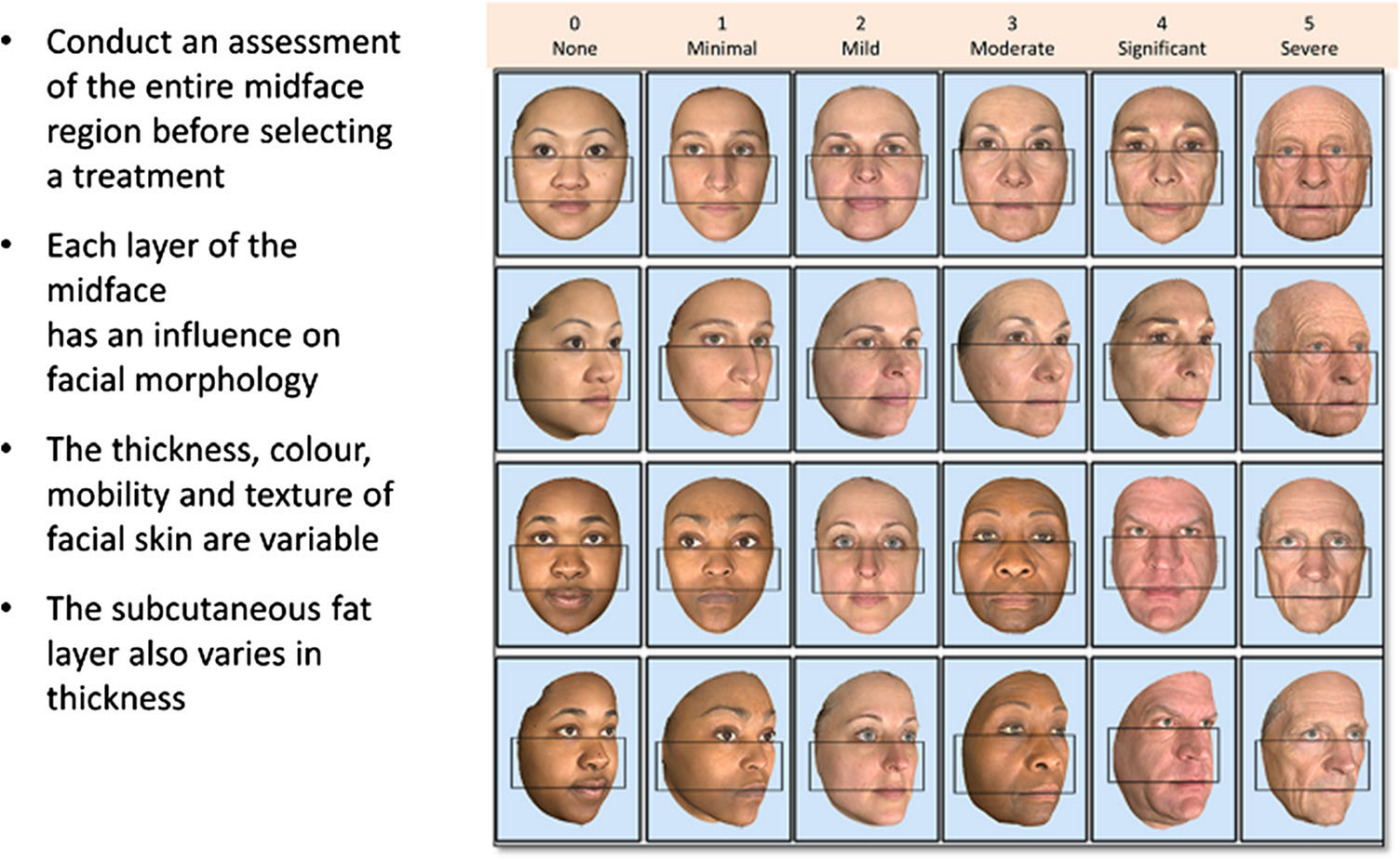
The Mid-Face Volume Deficit Scale was designed to evaluate the overall volume deficit of the mid-face, from 0 [none] to 6 or severe

The panel’s recommendation is that photographs should be taken at 0 degrees, at 45° (right and left) and at 90° (right and left). Additionally, it is thought to be extremely important to take dynamic photographs in order to detect asymmetries and the degree of skin sagging on neck flexion (Fig. 5).

### Procedure Care

#### Patient and Theatre Preparation

It is essential to standardize the surgical procedures to achieve the most desirable outcomes. In the panel’s opinion, there are different aspects that need to be taken into consideration when preparing the patient and the operating theatre for treatment. Table 3 shows the expert panel’s recommendations regarding these issues.

**Table 3:** Expert panel members’ recommendations for preparing the patient and the theatre for the aesthetic procedure

Recommendations for patient and material management
To have an auxiliary table
That helps us to maintain the aseptic conditions of the surgical materials
An assistant for opening the drawers
If not possible, it is mandatory to change or clean the gloves every time the surgeon touch something outside the sterile field
The patient bed should be foldable
Use of mask is recommended
Sterile gauzes should be opened starting the procedure*
It is recommended to leave them inside the plastic bag, especially when an assistant is not available
The physician should wash his/her hands thoroughly and remove their watch and rings
Patient must wash their hands with an alcohol-based gel hand disinfectant**
It is recommended to use sterile gloves
Always for cannulas
Recommended for needle***
Sharpen the pencils and clean them after each use
In order to avoid demarcation lines and skin pigmentation, it is mandatory to clean the skin that has been drawn before injecting
When needed, the lip must to be done at the end of the process
Surgeon must put special attention about do not touch any other part of the face after having touched the mucosa
If the surgeon needs to touch the face after handling the lip, it will be advisable to change the gloves

* Non-sterile gauzes can be used, but they should be damped with an antiseptic and kept on a clean surface throughout the procedure

** A specified quantity an alcohol-based gel hand disinfectant is placed in a subject’s cupped palms, and the subject rubbed the product onto all surfaces of the hands up to the wrists “until the patient felt their hands dry”

*** As syringes are not sterile, in such a case, the expert panel recommends to wear surgical gloves, although not necessarily sterile

#### Preparation of the Treatment Area

Although the incidence of infection following soft tissue injections is quite low [35–39], adequate skin preparation is critical to prevent superficial soft tissue infection [40]. Thus, patients with an ongoing skin infection in the area to be treated, or in close proximity, should not be treated [41]. Different commercially available antiseptic solutions have been used to prepare the skin for dermal filler procedures, yet no differences in terms of the rate of contaminated blood cultures were described with 10% povidone-iodine, 70% isopropyl alcohol, tincture of iodine, or povidone-iodine with 70% ethyl alcohol [40]. The panel recommends the combined use of a quaternary ammonium compound with 70% ethyl alcohol, or of chlorhexidine and 70% ethyl alcohol. However, it is important to note that chlorhexidine should be avoided in the periocular area due to the potential risk of keratitis and possible ocular damage [11].

There is no evidence that fillers trigger recurrent herpes infection, and thus, there is no reason to use anti-herpetic prophylaxis with every patient. Nevertheless, patients who have a history of developing cold sores after filler injection could benefit from such an approach. Hence, in patients with a history of recurrent herpes the panel recommends a prophylactic treatment with valacyclovir (1 g) for 3–4 days prior to treatment and for 3–4 days after.

Table 4 summarizes the panel’s recommendations regarding the preparation of the treatment area.

**Table 4:** Recommendations for preparing the area for filler administration

Preparing the area for filler administration
A surgical cap should be used
If it is not possible, at least, the hair should be correctly put up with a headband
It is highly recommended that the sink be located in the surgical room^a^
Clean the make-up properly^b^
Not to apply make-up since the day before the treatment.
If the patient comes made-up to the clinic, apply chlorhexidine at least for three minutes after removing the making-up
To use an antiseptic solution
The combination of a quaternary ammonium compound + 70% ethyl alcohol
The combination of chlorhexidine + 70% ethyl alcohol
Always disinfect all the skin and not only the area to be treated since most of the times, for example when handling cannulas, we touch different parts
To use disposable towels with antiseptic solution
They should be used before and after each injection at the injection point^c^
It is recommended to put antiseptic solution in the cover of the cannula
To regularly check that the needle is in perfect shape^d^
In terms of its sterile condition
To assess that it is not blunt
Do not refill the chlorhexidine bottles

^a^In some Spanish regions is mandatory

^b^As some kinds of make-up are indeed hard to remove in office, the panel recommends not to apply make-up since the day before the treatment

^c^This should never be a substitute of the antiseptic solution used before the procedure

^d^The panel recommends to change the needle after every 3–4 punctures

#### Anaesthesia

One of the main concerns of the patients with regard to the injection of the filler injections is the pain and discomfort associated, especially for the first-time patient. Whereas topical anaesthetics alone can provide adequate anaesthesia for some procedures, others require the injection of a local anaesthetic. However, while anaesthesia that blocks the nerves is effective against the pain, it may distort the area to be treated, as well as lengthen and complicate the procedure. Indeed, lidocaine injected in the infraorbital region distorts the local anatomy and may result in suboptimal correction [12]. Furthermore, there is a possibility that patients may experience an allergic reaction to lidocaine. However, a review of the literature showed that the incidence of true allergic reactions to local anaesthetic agents (including lidocaine and other products) was <1% [41].

#### Injection Technique

Selecting the appropriate technique for administration, based on the physician’s experience, the patient and the characteristics of the filler, helps to ensure successful outcomes and limits the incidence of complications. To identify and react to any adverse reaction, injections must be performed slowly and with caution. In terms of the filler, that amount administered should be adapted to each individual and injected in small quantities at multiple points to avoid overfilling [42]. Before injecting, aspiration should be performed as a prophylactic measure, particularly in highly vascularized areas, and a new needle without filler should be used prior to deep bolus injections [11].

For needle treatment, the panel recommends that adequate suctioning should be employed before injecting, at least 0.1–0.2 cc of air, and waiting for at least 4 s. Additionally, the experts also recommend changing the needle every 3 or 4 punctures. If the treatment is going to be administered using a blunt tip cannula, the panel recommends paying special attention to maintaining it sterile:

- The cannula should not be touched.
- Non-sterile areas should not be touched with the cannula.
- The cannula should be kept in its cap when not in use (it is recommended that antiseptic solution is put into the cannula cap).

### Post-procedural Care

Post-procedural care plays a vital role in achieving optimal results, and hence, the panel considers it is essential to inform patients as to how they should act after treatment.

Patients should be asked to comply with the following recommendations:

1. Avoid extreme cold or heat for 48 h.
2. Avoid massaging the treated area.
3. Avoid strenuous physical activity.
4. Sleep with their heads elevated for one night.
5. A skin care routine may be followed only once 24 h have passed.
6. Patients should not undergo dental procedures that might lead to gum bleeding in the 3–4 weeks following facial filler treatment.
7. In those patients with a predisposition to bruising, a vitamin K cream or arnica gel may be useful to speed up its resolution.

#### Documentation

To trace untoward effects that might arise from manufacturing discrepancies, the batch number sticker provided with the filler should be attached to the patient’s consent form or medical chart. Indeed, the entire process should be exhaustively documented, including the injection pattern and technique, the name and amount of the filler injected, and the area(s) treated. The panel considers it extremely useful to use a diagnostic/treatment code tool.

## Conclusions

Aesthetic medical procedures with dermal fillers are becoming increasingly popular. For this reason, aesthetic clinicians must become aware of how to prevent and how to manage any potential complications. The panel agrees that the adequate selection of the patient, technique and filler will help to ensure a desirable outcome. Moreover, to prevent future undesirable outcomes and serious adverse events, it will be important to carefully document the procedure, technique and the filler administered. A diagnostic/treatment code tool may be suitable to achieve this. This consensus highlights key elements that will help clinicians who are just starting to use dermal filler procedures, and it could serve as a basis to standardize the process and to establish how to prevent potential complications of this treatment.
